# Impact of Macroscopic On-Site Evaluation (MOSE) on Accuracy of Endoscopic Ultrasound-Guided Fine-Needle Biopsy (EUS-FNB) of Pancreatic and Extrapancreatic Solid Lesions: A Prospective Study

**DOI:** 10.3390/diagnostics12020428

**Published:** 2022-02-07

**Authors:** Silvia Gaia, Stefano Rizza, Mauro Bruno, Davide Giuseppe Ribaldone, Francesca Maletta, Marco Sacco, Donatella Pacchioni, Felice Rizzi, Giorgio Maria Saracco, Sharmila Fagoonee, Claudio Giovanni De Angelis

**Affiliations:** 1 Gastroenterology, AOU Città della Salute e della Scienza di Torino, 10126 Turin, Italy; stefanorizza@live.it (S.R.); mabru1964@gmail.com (M.B.); davrib_1998@yahoo.com (D.G.R.); marco.sacco10@gmail.com (M.S.); rizzifelice91@libero.it (F.R.); giorgiomaria.saracco@unito.it (G.M.S.); eusdeang@hotmail.com (C.G.D.A.); 2 Pathology Unit, Department of Laboratory Medicine, AOU Città della Salute e della Scienza di Torino, 10126 Turin, Italy; fmaletta@cittadellasalute.to.it (F.M.); dpacchioni@cittadellasalute.to.it (D.P.); 3 Molecular Biotechnology Center, Institute of Biostructure and Bioimaging (CNR), 10129 Turin, Italy; sharmila.fagoonee@unito.it

**Keywords:** EUS, FNB, MOSE

## Abstract

This is a prospective and comparative study including 76 consecutive patients performing EUS-FNB for pancreatic and extrapancreatic solid lesions, randomized by alternate allocation to macroscopic on-site evaluation (MOSE) (40 patients) or to a conventional technique (40 patients), with three passes each. MOSE samples were differentiated into score 0: no visible material, score 1: only necrotic or haematic material, score 2: white core tissue ≤ 2 mm, or score 3: white core tissue > 2 mm. The conventional technique consisted in pushing all the needle content into a test tube for evaluation by the pathologist. In both groups, a 22–25 Gauge Franseen-tip needle (Acquire, Boston Scientific Co., Natick, MA, USA) was used. The study evaluated the diagnostic accuracy and adequacy of MOSE compared to the conventional technique and whether MOSE could optimize the number of passes during EUS-FNB. **Results**: The analysis was performed on 76 patients (38 MOSE, 38 conventional). The overall diagnostic adequacy was 94.7% (72/76) and accuracy was 84.2% (64/76). The diagnostic accuracy was similar in the two groups: MOSE 86.8% (33/38 lesions), vs. conventional 81.6%, 31/38 lesions, *p* = 0.76). Regarding diagnostic adequacy, the MOSE technique was 97.4% (111/114 passes) compared to 92.1% (105/114 passes) with the conventional technique, *p* = 0.06. The accuracy increased according to the MOSE score evaluation: it was 43.5%, 65.5% and 78.3% in patients with score 1, score 2, and score 3, respectively. Moreover, if in the first two passes the MOSE score was 2 or 3, the accuracy was 82.6% (20/23), and upon adding a third pass, the accuracy increased to 87% (20/23), which was not significantly different from the general accuracy of the MOSE samples (86.8%) (*p* = 0.86). **Conclusions**: The MOSE score showed a comparable diagnostic accuracy to the conventional technique. However, MOSE allows endoscopists to perform an inspective evaluation of the material, tends to perform better than the conventional technique in terms of diagnostic adequacy, and may potentially reduce the number of passes.

## 1. Introduction

The endoscopic ultrasound-guided fine-needle biopsy (EUS-FNB) is potentially capable of providing sufficiently large and well-preserved core specimens to allow for the assessment of the architecture of the surrounding tissue and stroma, and it has been proposed as an alternative to overcome the limits of FNA [1,2,3,4,5]. Macroscopic on-site evaluation (MOSE) consists in the direct macroscopic evaluation of the core tissue obtained from EUS-FNB by the operator, in order to estimate the adequacy of the sample to be provided for subsequent cyto-histological evaluation [6].

Currently, the potential of MOSE, compared to conventional tissue acquisition methods, still remains uncertain. In theory, the advantage of this approach lies in the fact that the endosonographer can have an immediate idea, “on-site”, of the material taken in terms of quantity and quality—data that may potentially be useful when deciding the number of passes. This could be especially useful in patients with a higher procedural risk, or for whom a shorter examination duration and/or less invasiveness can constitute an element of safety.

The aim of the present study was to evaluate the accuracy of the MOSE technique and its diagnostic adequacy compared to the conventional technique during the FNB of pancreatic and extrapancreatic lesions. Moreover, the study evaluated whether the MOSE technique could reduce the number of passes by providing a macroscopic evaluation of the sample.

## 2. Patients and Methods

This is a prospective and comparative study, performed at the Gastroenterology and Digestive Endoscopy, third level endoscopy centre of the University Hospital “Città’ della Salute e della Scienza” in Turin, Italy, between December 2019 and September 2020. We obtained written informed consent from all patients after the study protocol was approved by local Ethics Committee (Number of protocol CS/643). The study was performed according to the principles of the Declaration of Helsinki (1989). The study design followed the CONSORT statement rules (www.consort-statement.org) (accessed on 3 February 2022).

All consecutive patients (aged > 18 years) referred for EUS-FNB of solid pancreatic or extrapancreatic lesions (lymphadenopathy, subepithelial lesions, and others) were included. The exclusion criteria were coagulopathy (INR > 1.5, platelets count < 50,000/L), altered anatomy of the upper digestive tract (e.g., previous Billroth II operation, Roux-en-Y anastomosis), anaesthetic contraindications, or cystic lesions. Pregnant patients and those unable to provide informed consent were also excluded.

In patients who fulfilled the inclusion criteria, EUS-FNB was performed, and the sample was alternately randomized for evaluation with a conventional technique or with MOSE (conventional-FNB alternate to MOSE-FNB).

### 2.1. EUS-FNB Procedure

The procedure was performed by three expert endosonographers (SG, MB CD > 150–200 EUS per year) using a linear echoendoscope (GF-UCT 180, Olympus Co., Ltd., Tokyo, Japan). Procedures were performed with anaesthesiologic support.

The target lesion was evaluated by B-mode, eco-colour-power Doppler and, if indicated, by SonoVue contrast medium (Bracco Imaging Italia, Milan, Italy). FNB was performed using a 22–25 Gauge Franseen-tip needle (Acquire, Boston Scientific Co. Ltd., Natick, MA, USA). Each target was sampled with three passes, using the “slow pull” or “suction” by performing 10–20 strokes within the lesion, as recommended by the ESGE guidelines [7].

More specifically, the endosonographers started with the “slow pull” technique and, in the case of “punctio sicca”, the operator could decide to change the method by performing a “suction”. These two techniques have been described to be similar in terms of samples acquisition [8] and are both commonly used in our center. This study follows what is usually performed in clinical practice. The “slow pull” technique consists of slowing the retraction of the stylet during the sampling; the “suction” technique consists of affixing a 10 mL suction syringe to the needle.

If none of the three passes obtained sufficient material, or were deemed unsatisfactory, further passes were carried out at the endoscopist’s discretion with a different technique and needle type, and were not included in the present study. After FNB, patients were observed for immediate adverse events for at least 4 h.

### 2.2. Sample Management

**Figure 1:**
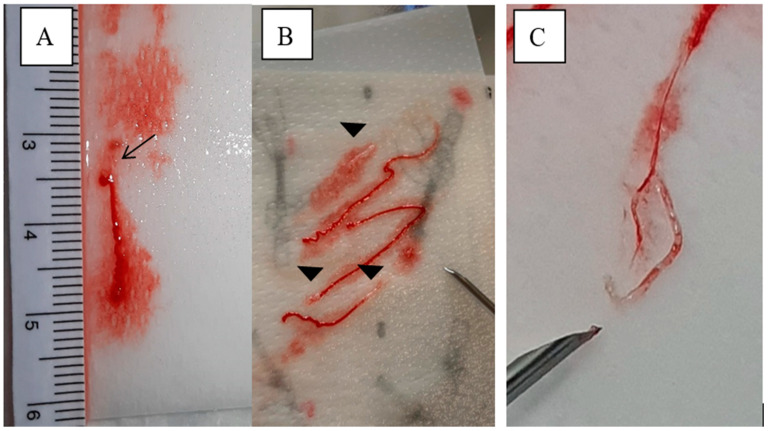
**Representative samples scored with MOSE technique.** (**A**) MOSE score 2, single < 2 mm white sample (arrow) with hematic material. (**B**) MOSE score 3, multiple, >2 mm yellowish-white material (arrowheads). (**C**) MOSE score 3, single long white material.

**MOSE-FNB:** The content of the needle was swiped on filter paper [8], moistened with saline solution, and inspected by the operator to assess the presence of tissue core clearly visible according to the MOSE technique. The sample was examined with a ruler by a visual scale and classified as shown in Table 1 and Figure 1.

Filter paper containing the streaked material was folded, placed inside a cassette, and stored in test tubes filled with formalin-based solutions and sent for histological analysis.

**Figure 2:**
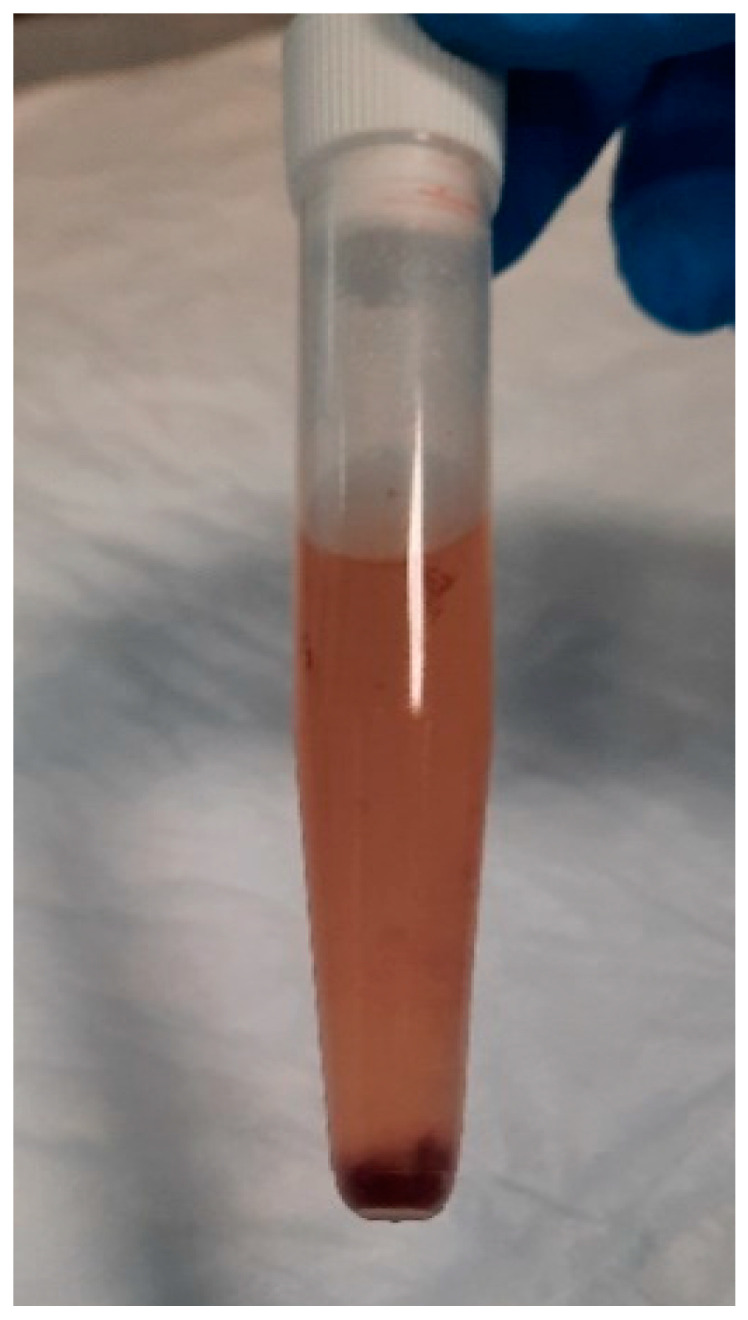
Conventional technique. Sample is flushed in a test tube containing alcoholic solution.

**Conventional-FNB:** All the content of the needle was pushed by the stylet, flushed by saline into a test tube containing 95% alcohol (Figure 2), and sent to the pathology laboratory.

### 2.3. Cyto-Histological Evaluation

Two dedicated pathologists evaluated the samples obtained in terms of quantity, quality and blood contamination, and provided a cyto-histological diagnosis.

In the “MOSE-FNB group”, the samples were processed in the filter paper and embedded in paraffin in tissue-blocks as small biopsy specimens. In the “conventional-FNB” group, the samples collected in tubes with 95% alcohol were centrifuged: pellets obtained from centrifugation were paraffin-embedded in cell-blocks. For both types of processing, 3–4 µm sections were obtained from tissue and cell-blocks, and stained with conventional staining (Hematoxylin and Eosin, Papanicolaou stain). Moreover, for both types of processing, further sections could be obtained for immunohistochemical or molecular analyses, if necessary.

### 2.4. Gold Standard for Diagnosis

The gold standard for the final diagnosis was histological examination of the surgical sample in cases where the patient’s clinical history led to surgery with removal of the lesion itself, or metastases if biopsied in further evaluations. If no surgical indication was found, a minimum clinical and imaging follow-up time of 9 months was respected to observe the disease course, and to thereafter confirm the results of the EUS-FNB.

### 2.5. Outcomes

The primary aim was to compare the accuracy of the MOSE technique and conventional technique in FNB of solid pancreatic and extrapancreatic lesions. The overall diagnostic accuracy was defined as the proportion of correct diagnoses.

The secondary aims were to evaluate whether the MOSE technique can reduce the number of passes during FNB, providing macroscopic sample examination. The diagnostic accuracy of each single pass, as well as the concordance between the MOSE score and the pathologist’s judgment were assessed.

The overall diagnostic adequacy was defined as the rate of cases in which a specific histological diagnosis was rendered. For practical purposes, specific diagnoses were grouped into two categories, namely, benign and malignant, the latter encompassing neoplasms of different malignant potential, for example, adenocarcinoma, neuroendocrine tumors and lymphoma. Moreover, the sensitivity, specificity, positive predictive value (PPV), negative predictive value (NPV), and rate of complications related to the EUS-FNB procedure were evaluated.

### 2.6. Statistical Analysis

Continuous data were presented as mean and standard deviation or as a median with interquartile range, according to their distribution. Means were compared using Student’s *t*-test and medians were compared using Mann–Whitney’s U-test, which is a test for independent samples of non-normally distributed variables. Categorical variables were presented as percentage; Pearson’s or Fisher’s exact test was used for comparisons, while 95% confidence intervals (CI) of the percentages were calculated using the normal approximation. A *p*-value of less than 0.05 was considered statistically significant. To determine the sample size, the type I error and power were considered (0.05 and 80%, respectively). The sample size was calculated on the basis of literature data regarding accuracy: 98.3% for MOSE [9] and 79.1% for conventional FNB [10]. A power level of 80% with a significant value of ≤ 0.05 (two-sided Fisher’s exact test) required a sample size of 40 patients for each group.

Per protocol analysis was performed. All statistical analyses were performed using MedCalc version 18.9.1.

## 3. Results

**Figure 3:**
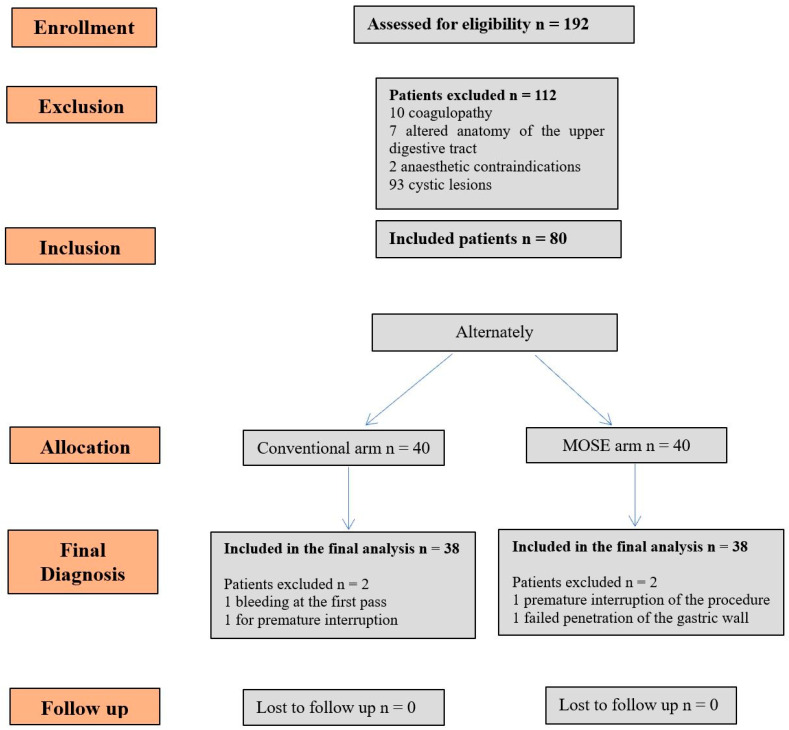
Flow-chart of the study.

The flow-chart in Figure 3 reports the details of the present study. Eighty patients fulfilled the criteria and were enrolled in the study: 40 in the MOSE arm and 40 in the conventional arm. In the MOSE arm, two patients were excluded from the final cyto-histological analysis as it was not possible to follow the established protocol—in one case for premature interruption of the examination due to anaesthesiologic problems, and in the other for failed penetration of the gastric wall. In the conventional arm, two patients were excluded: one for evidence of bleeding at the first pass which necessitated halting the procedure, and one for premature interruption of the examination due to anaesthesiologic problems. Per protocol analysis was performed: a total of 76 patients were included in the final analysis, and none of them was lost at follow-up. Features of patients and lesions included in the study are reported in Table 2. There were no statistically significant differences between the two arms in terms of gender, mean age, mean lesion size, and indication for EUS-FNB.

In 24 cases (31.6%) (12 in the MOSE group and 12 in the conventional group), the final diagnosis was defined after surgical operation, while in 52 cases (26 in the MOSE group and 26 in the conventional group), it was based on clinical-instrumental follow-up for a minimum of 9 months. Final diagnoses for both groups are reported in detail in Table 3 (also including the four cases excluded from established protocol).

The mean number of passes, considering the extra ones beyond the three established in the protocol, was 3.8 (95% CI: 3.5–4) in the MOSE arm vs. 3.7 (95% CI: 3.4–3.9) in the conventional arm (*p* = non-significant).

Extra passes, no more than two, were overall performed in 38/76 cases (50%), respectively in 16/38 cases (42.1%) for the MOSE arm and in 22/38 (57.9%) cases for the conventional arm.

### 3.1. Adequacy and Diagnostic Accuracy of MOSE Compared to Conventional FNB

The overall diagnostic adequacy was 94.7% (72/76) and accuracy was 84.2% (64/76). There was no statistically significant difference between the two arms in terms of diagnostic accuracy (MOSE 86.8% vs. conventional 81.6%, *p* = 0.76). Diagnostic adequacy with the MOSE technique was 97.4% (111/114 passes) compared to 92.1% (105/114 passes) with the conventional technique, *p* = 0.06.

**Figure 4:**
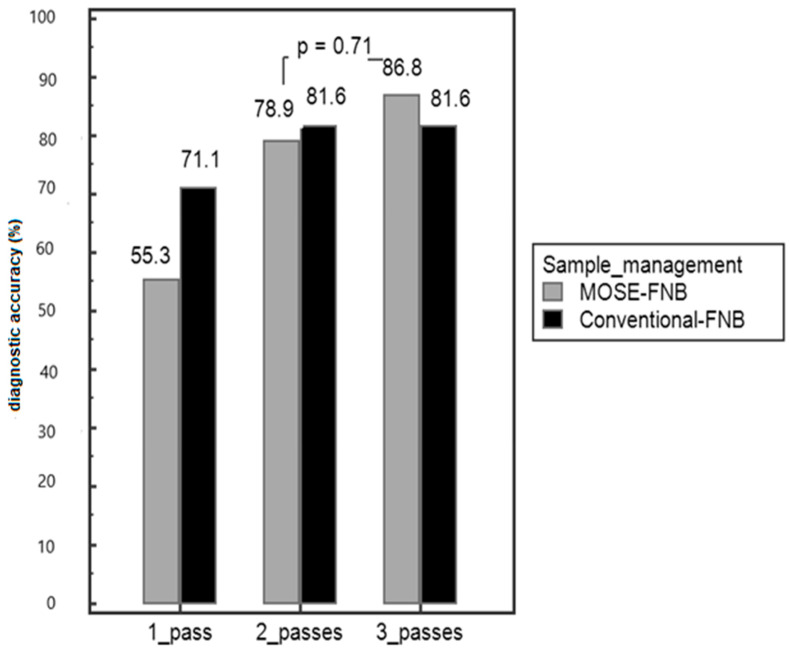
Diagnostic accuracy of each single pass.

There was no difference between the two techniques in terms of negative predictive value (NPV) and positive predictive value (PPV), as shown in Table 4. In Figure 3, the diagnostic accuracy of each single pass in MOSE and conventional FNB is shown (Figure 4).

Each MOSE sample was scored from 1 to 3 based on the macroscopic aspect to assess the presence of clearly visible clots or tissue cores (yellowish-white or pink), (Table 1 and Figure 1).

Among samples with a MOSE score of 1, the accuracy was 43.5% (10 correct diagnoses out of 23); among those with a MOSE score of 2, the accuracy was 65.5% (20 correct diagnoses out of 31); and in cases with a MOSE score of 3, the accuracy was 78.3% (47 correct diagnoses on 60 samples), as reported in Table 5. Moreover, if in the first two passes the MOSE score was 2 or 3, the accuracy was 82.6% (19 out of 23). By adding a third pass, the accuracy increased to 87% (20/23), *p* = 0.87 but was not significantly different from the overall accuracy of the MOSE technique (86.8%, *p* = 0.86).

### 3.2. Procedure-Related Complications

Two cases (2.6%) of intra-procedural bleeding were reported: one patient showed bleeding after the first pass of the needle which led to interruption of the procedure, while another patient had slight blood dripping, self-resolved without the need for hospitalization or blood transfusions. A single case of mild oedematous acute pancreatitis requiring hospitalization was reported. The total major complication rate was 3.8% (3/80).

Among minor complications observed, an episode of fever in the hours following the procedure was registered in one patient, without any indication for infectious investigations, and which resolved in a few days without sequelae.

## 4. Discussion

The ability to obtain an adequate sample for diagnosis with a smaller number of passes is of paramount importance in EUS-guided tissue acquisition. With the MOSE technique, the endosonographer can macroscopically examine the sample and describe the type of collected material, the presence of blood or necrosis, or absence of material. In this study, we reported how the MOSE technique had excellent adequacy (98%) and good accuracy (87%), which may help in reducing the number of passes and increasing consciousness of the type of collected sample.

We report herein the results of comparison between EUS-FNB with the MOSE and conventional techniques. The most relevant finding is that the MOSE technique has the same accuracy and adequacy compared to the conventional technique, but when MOSE is used, a macroscopic core is visible, and the accuracy increases compared to when only a clot sample is visible. When the material is swiped on filter paper, the operator can have an immediate perception of its quantity and quality. The accuracy increased from 43% with a MOSE score of 1 (only clot), to 65% with a score of 2 (2 mm core) and 78% with a score of 3 (more than one and beyond 2 mm macroscopic cores visible).

Our study demonstrates that if the first two passes give a score of 2 (core < 2 mm) or 3 (>2 mm), the probability to have a final diagnosis is high (accuracy 83%) and the endosonographer can stop the procedure, thus avoiding the third pass (accuracy 87%, *p* = non-significant). In fact, in this case, the accuracy rate is not significantly different from the overall accuracy obtained in this study (84%) or with the conventional technique (81%). These data are of prime importance in the case of high-risk patients in terms of complications or anaesthesiologic compliance, as by reducing the number of passes, the procedure time is shortened. In our study, the complication rate was similar to that reported in the literature. Three patients (3.8%) had major complications, such as bleeding or acute pancreatitis. As each pass can increase the risk of complications, reducing the procedure time and the number of passes may help in decreasing the complication rate.

The overall accuracy of 84% (86.8% MOSE vs. 81.8% conventional) was slightly below the most recent evidence relating to the diagnostic accuracy of EUS-FNB needles, which would be greater 90% (compared to 80% of the EUS-FNA). Nevertheless, our data are in agreement with the reported range of diagnostic accuracy of the EUS-guided tissue acquisition, oscillating between 50% and 98%. Second-generation FNB needles are increasingly used to obtain histological tissue samples and, as shown by a meta-analysis of 15 studies including 1024 patients, these can achieve a high diagnostic yield [4].

Regarding the diagnostic adequacy, no significant differences were observed between the two arms (97.4% MOSE vs. 92.1% conventional, *p* = 0.06) with the limitation of a small number of patients enrolled in this study. However, the overall adequacy (94.7%) was in line with literature data: the diagnostic adequacy of Acquire needles ranged between 98% and 100% [5,9,10].

The MOSE score proposed in this study considered the small core samples of 2 mm, and it showed that these small fragments of tissue can also have an appreciable role in the diagnosis (accuracy of 65%). Iwashita et al. [2] proposed a MOSE score with a bigger mascroscopic visible core, suggesting that a core of >4 mm showed significantly superior histologic, cytologic, and overall diagnostic yields; however, in this study, 19G needles were used.

Recent studies [11,12] by Kaneko et al. showed that visible cores exceeding 10 mm, using a 22-gauge Franseen needle for EUS-FNB, may be useful for the correct diagnosis of pancreatic masses. However, in our experience, it is not always possible to obtain >10 mm cores, and the present study included both 22G and 25G needles. The 25G needles are commonly used in our clinical practice to adapt to different types of situations (such as in highly vascularized tumour or risk of haemorrhage for interposed vessels), and they are known to obtain thinner and smaller samples. The macroscopic evaluation scale proposed in this study had a good correlation with the pathologists’ judgment: in almost all passes (97.4%), there was agreement between MOSE and pathological adequacy. The cases defined as “non-diagnostic” were mainly from passes on pancreatic adenocarcinomas. These lesions tend to trigger an intense non-neoplastic reaction of the host pancreas consisting of fibroblasts, lymphocytes, and extracellular matrix (“desmoplastic” reaction) and may present areas of particular degeneration containing necrotic material. These areas, due to the scarce presence of cellularity typical of adenocarcinoma, tend to reduce or weaken the diagnostic yield of the needle pass. Macroscopic evaluation may confuse necrotic tissue (whitish) with solid lesion tissue even if necrosis is flaking off more. This is a fact to be taken into consideration when MOSE is performed, and it may explain why, in our study, the accuracy in a MOSE score of 2 or 3 was slightly lower than those reported in other studies [11,12,13]. Considering the concordance between the MOSE score and pathological judgement of adequacy, it is important to note that necrosis cannot be easily differentiated from an adequate material. However, despite the similar colour, the consistency is different: necrosis is more crumbling with a melting texture compared to a tissue core. Moreover, sometimes, clots may contain small tumoral cells that cannot be macroscopically evidenced; these data explain why in a MOSE score of 1, the accuracy was 43.5%.

In a recent multicentre study by Chong et al. [14], MOSE proved to be a reliable technique in the absence of ROSE, giving a diagnostic yield comparable to the conventional technique but with fewer passes. However, since the new-generation FNB needles were not yet widespread in clinical practice, the needle used was a 19 Gauge, which is often difficult to use in some locations. In our study, on the other hand, finer needles such as 22 and 25 Gauge were used. In the monocentric study by Leung et al. [15], the MOSE technique was tested on smaller Gauge needles. An Acquire 22 Gauge FNB needle was used with the standard aspiration technique, and the authors did not perform further passes in the presence of at least one light pink–brown core tissue, regardless of its length, following rough visual inspection. In 93% of cases, this result was obtained with a single pass, while in 4/54 cases, a second pass was required, with an overall diagnostic adequacy and accuracy of >90%. Several recent studies increasingly support the importance of MOSE and its contribution in terms of diagnostic yield and accuracy [16,17,18,19].

In general, FNB needles possess the ability to supply a greater quantity of tissue with preserved architecture, hence further opening the door to the possibility of performing molecular analyses on the tissue, and supporting the concept of “targeted therapy”. According to the experience of our pathologists, the core tissue macroscopically evaluated with MOSE and fixed in formalin for histology is better suited than traditional cytology in absolute alcohol for searching molecular alterations, potential chemotherapeutic targets, such as the PD-L1 protein (Programmed Death-Ligand 1).

Our study has some limitations, such as the small number of patients enrolled relative to a single centre and the small number of lesions subjected to FNB per single subtype, and the lack of molecular profiling. Nevertheless, the follow-up was longer than 9 months in average compared to other studies. Larger studies are required to further support our findings.

In conclusion, our study corroborates that FNB performed with MOSE is an easy and safe technique, which allows for an immediate, preliminary visual evaluation of the material obtained in order to supply adequate material for diagnosis after fewer passes. This offers significant advantages, such as reduction in the number of passes and risk of related complications.

## Figures and Tables

**Table 1:** MOSE score and sample classification.

MOSE Score	Contents of the Biopsy	Classification of the Sample
0	punctio sicca/no material	negative (−)
1	only necrotic or haematic material	acceptable (+)
2	≥1 core tissue ≤ 2 mm yellowish-white	positive (+)
3	≥1 core tissue > 2 mm yellowish-white	positive (+)

**Table 2:** Baseline characteristics of the patients and lesions.

	MOSE (n = 38)	Conventional (n = 38)	*p* Value
**Male sex, n (%)**	24 (63.2%)	23 (60.5%)	0.81
**Age, mean (SD), years**	67.2 (9.95)	69.9 (11.2)	0.59
**Size of lesion, median (IQR), mm**	31.5 (25–40)	30.5 (20–40)	0.54
**Indication, n (%)**			
**-Pancreatic lesion**	24 (63.2%)	25 (65.8%)	
**-Lymphadenopathy**	7 (18.4%)	7 (18.4%)	
**-Subepithelial lesion**	4 (10.5%)	5 (13.2%)	
**-Others**	3 (7.9%)	1 (2.6%)	
**Mean number of needle passes (SD)**	3.8 (0.84)	3.7 (0.61)	0.46
**FNB site**			
**-Esophagus**	4 (10.5%)	3 (7.9%)	
**-Stomach**	22 (57.9%)	22 (57.9%)	
**-Duodenum**	12 (31.6%)	13 (34.2%)	

SD = standard deviation; IQR = interquartile range.

**Table 3:** Final diagnoses in MOSE ad Conventional arms.

MOSE Arm	Type of Lesion
**Pancreatic lesions (24)**	17 ductal adenocarcinoma
	3 chronic pancreatitis
	2 neuroendocrine tumor (NET)
	1 acinar carcinoma with NET foci
	1 regular pancreas
**Extrapancreatic lesions (16)**	7 neoplastic/metastatic lymph node
	4 stromal lesions (GIST/leiomyoma)
	2 inflammatory lymph node
	2 ampullary adenocarcinoma
**CONVENTIONAL Arm**	
**Pancreatic lesions (25)**	20 ductal adenocarcinoma
	3 neuroendocrine tumor (NET)
	2 chronic pancreatitis
**Extrapancreatic lesions (15)**	5 neoplastic/metastatic lymph node
	2 stromal lesion (GIST/leiomyoma)
	2 inflammatory lymph node
	2 cholangiocarcinoma
	1 squamous cell carcinoma of the oesophagus
	1 carcinosarcoma
	1 undifferentiated gastric adenocarcinoma
	1 vascular malformation with calcifications

**Table 4:** Diagnostic performance with two passes in the MOSE and conventional techniques.

	MOSE (n = 38)	Conventional (n = 38)	*p* Value
**Diagnostic adequacy**	79%	82.2%	>0.05
**Sensitivity**	78%	81.50%	>0.05
**Specificity**	100%	100%	>0.05
**PPV**	100%	100%	>0.05
**NPV**	41.6%	41.9%	>0.05
**Diagnostic** accuracy	79%	82.2%	>0.05

**Legend:** PPV: positive predictive value; NPV: negative predictive value.

**Table 5:** Comparison between MOSE score for each single pass and adequacy of the sample.

MOSE Score	N Samples	Adequacy (%)	Accuracy (%)
0—negative	**0**	0 (0%)	0
1—acceptable	**23**	23 (100%)	10 (43.5)
2—positive	**31**	29 (93.5)	20 (65.5)
3—positive	**60**	59 (98.3)	47 (78.3)
**Total**	**114**	111/114 (97.4)	77/114 (67.5)

## Data Availability

The data that support the findings of this study are available on request from the corresponding author.
